# Codon usage suggests that translational selection has a major impact on protein expression in trypanosomatids

**DOI:** 10.1186/1471-2164-9-2

**Published:** 2008-01-03

**Authors:** David Horn

**Affiliations:** 1 London School of Hygiene & Tropical Medicine, Keppel Street, London, WC1E 7HT, UK

## Abstract

**Background:**

Different proteins are required in widely different quantities to build a living cell. In most organisms, transcription control makes a major contribution to differential expression. This is not the case in trypanosomatids where most genes are transcribed at an equivalent rate within large polycistronic clusters. Thus, trypanosomatids must use post-transcriptional control mechanisms to balance gene expression requirements.

**Results:**

Here, the evidence for translational selection, the enrichment of 'favoured' codons in more highly expressed genes, is explored. A set of highly expressed, tandem-repeated genes display codon bias in *Trypanosoma cruzi*, *Trypanosoma brucei *and *Leishmania major*. The tRNA complement reveals forty-five of the sixty-one possible anticodons indicating widespread use of 'wobble' tRNAs. Consistent with translational selection, cognate tRNA genes for favoured codons are over-represented. Importantly, codon usage (Codon Adaptation Index) correlates with predicted and observed expression level. In addition, relative codon bias is broadly conserved among syntenic genes from different trypanosomatids.

**Conclusion:**

Synonymous codon bias is correlated with tRNA gene copy number and with protein expression level in trypanosomatids. Taken together, the results suggest that translational selection is the dominant mechanism underlying the control of differential protein expression in these organisms. The findings reveal how trypanosomatids may compensate for a paucity of canonical Pol II promoters and subsequent widespread constitutive RNA polymerase II transcription.

## Background

Trypanosomatids have a devastating impact on the world's poor, causing African trypanosomiasis, Chagas disease and leishmaniasis [1]. The consequences of this range of human and animal diseases are hundreds of thousands of deaths each year, ~1.5 million cases a year of the disfiguring lesions associated with cutaneous leishmaniasis and severely curtailed agricultural development throughout sub-Saharan Africa. The African trypanosome also causes Nagana disease in cattle, rendering 10 million square kilometres of land unsuitable for livestock.

The protozoan parasites responsible branched early from the eukaryotic lineage and display a range of unusual molecular features. RNA polymerase II transcription of protein coding genes is polycistronic and constitutive and all mature mRNAs are trans-spliced to an identical leader sequence [2]. Genome sequencing revealed remarkably conserved gene order or synteny across the genomes of the African trypanosome, *Trypanosoma brucei*, the South American trypanosome, *Trypanosoma cruzi*, and *Leishmania major *[3]. These trypanosomatids cause distinct diseases, are spread by different insect vectors and are thought to have diverged from a common ancestor several hundred million years ago. Trypanosomatids display a unique paucity of conventional RNA polymerase II promoters and transcriptional control is extremely limited compared to any other organism studied in any detail. Widespread constitutive and polycistronic transcription places considerable emphasis on post-transcriptional control since genes in the same transcriptional cluster function in unrelated pathways and are expressed at widely different levels [4]. Thus, trypanosomatids present a unique opportunity to study post-transcriptional control of gene expression.

Cells must express different proteins over an enormous abundance-range, from fewer than 50 to more than a million molecules per cell reported in *Saccharomyces cerevisiae *[5]. Efficiently translated mRNA species are translated several thousand times, with translation initiating up to once every 2 s, providing substantial scope for differential control at the level of translation. Translational selection has been reported in a range of species, whereby more frequent synonymous codons correspond to more abundant cognate tRNAs, with the correspondence being more pronounced for highly expressed genes [6-8]. Synonymous codon bias has also been reported in trypanosomatids [9-12]. There is a good correlation between mRNA levels and protein levels in yeast with 73% of variance in protein abundance explained by mRNA abundance [13]. In contrast, microarray analysis reveals modest differences in mRNA abundance in trypanosomatids and proteome analysis suggests substantial differential control at the level of translation or protein turnover [14-17]. Evidence for translational selection is explored here using trypanosomatid genome sequence data [18-20] and whole-cell proteome data [21]. The findings suggest that translational selection is the dominant mechanism underlying the control of differential protein expression in trypanosomatids.

## Results

### Tandem genes are highly expressed and display codon bias

Most trypanosomatid genes are 'single copy' (trypanosomatid genomes are typically diploid) but tandem gene amplification is thought to contribute to increased expression [20,22]. Thus, tandem amplified genes may be among the most highly expressed and, if translational selection operates, may be an excellent source of favoured codons. To begin to explore evidence for translational selection in trypanosomatids, codon bias was assessed in tandem amplified genes. Whole-cell proteome data has been derived from *T. cruzi *[21] but the genome assembly is incomplete due to sequence complexity; the strain used for the sequencing project is a hybrid of two genotypes with multiple distinct alleles for most genes [3]. Since the *T. brucei *assembly is excellent and most trypanosomatid genes share orthologues accessible through the GeneDB interface, the *T. brucei *genome was scanned for tandem duplicated protein-coding genes. Consistent with the first prediction above, sixty-four tandem-amplified genes in *T. brucei *encode proteins with orthologues among the 243 proteins over-represented (≥ 10 mass spectra) in the non-redundant *T. cruzi *proteome set [21]. This includes the histones, ribosomal proteins, chaperones, tubulins and enzymes of carbohydrate metabolism. Tandem arrayed genes display little or no sequence divergence so a single copy from each tandem was selected for further analysis (see Table 1 in Additional file 1).

**Figure 1:**
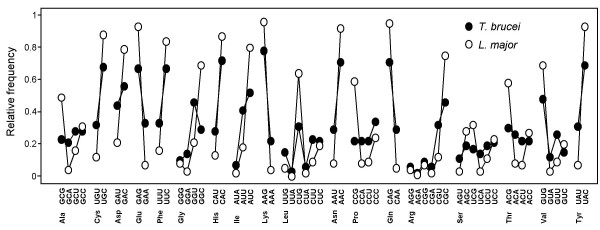
Relative frequency of synonymous codon usage in the tandem, high expression gene sets from *T. brucei *and *L. major*; codon usage patterns were broadly similar in *T. brucei *and *T. cruzi *(data not shown). Tandem amplified genes were considered highly expressed if represented by ≥ 10 mass spectra from whole-cell proteome analysis of four life-cycle stages of *T. cruzi *[21].

Codon usage was analysed for this tandem gene set from *T. brucei *and for the orthologous sets from *T. cruzi *and *L. major*, >60,000 codons in total. Consistent with previous reports [9,11,12], this revealed codon bias in all three trypanosomatids (Table 1). An extreme example is the gene encoding the highly abundant α-tubulin gene in *L. major *which uses only 40 of the 61 available codons. Figure 1 illustrates this bias across all synonymous codons for *T. brucei *and *L. major*. What is clear from Figure 1 is that codon bias is more pronounced in *L. major*. This is likely explained by the higher 'background' GC-content; the intergenic and protein-coding GC-contents are 41% and 50.9% in *T. brucei *[18], 47% and 53.4% in *T. cruzi *[19] and 57.3% and 62.5% in *L. major *[20] respectively. Since RNA sequences probably compete for access to the translation machinery and most GC3-codons (codons with G or C at the third position) are favoured, the higher GC-content may have driven the increase in codon bias within protein coding regions.

**Table 1:** Codon usage across the trypanosomatid tandem gene set (see table 1 in the additional data file, >60,000 codons) and tRNA gene copy number (see table 2 in the additional data file) are shown. Overall favoured codons and all sixteen CU3 'wobble tRNA pairs' are indicated in bold text.    *T. cruzi *has additional tRNA genes in many cases because the strain used for the sequencing project is a hybrid of two genotypes [19].

**Am Acid**	**Codon**	***T. brucei *Number**	**Fraction**	**tRNAs**	***T. cruzi *Number**	**Fraction**	**tRNAs**	***L. major *Number**	**Fraction**	**tRNAs**
**Ala**	**GCG**	452	0.24	2	685	0.36	2	925	0.49	2
**Ala**	GCA	406	0.21	1	283	0.15	1	79	0.04	1
**Ala**	GCU	526	0.28	**2**	325	0.17	**2**	299	0.16	**2**
**Ala**	GCC	516	0.27	**0**	629	0.33	**0**	593	0.31	**0**
										
**Arg**	AGG	93	0.07	1	95	0.07	2	45	0.04	1
**Arg**	AGA	29	0.02	1	25	0.02	2	15	0.01	1
**Arg**	CGG	117	0.09	1	103	0.08	2	86	0.07	1
**Arg**	CGA	82	0.06	1	55	0.04	2	21	0.02	1
**Arg**	CGU	415	0.31	**3**	295	0.23	**4**	148	0.12	**4**
**Arg**	**CGC**	592	0.45	**0**	730	0.56	**0**	922	0.75	**0**
										
**Asn**	AAU	248	0.29	**0**	178	0.23	**0**	64	0.08	**0**
**Asn**	**AAC**	596	0.71	**2**	597	0.77	**4**	716	0.92	**3**
										
**Asp**	GAU	501	0.44	**0**	293	0.27	**0**	216	0.21	**0**
**Asp**	**GAC**	639	0.56	**2**	795	0.73	**2**	804	0.79	**3**
										
**Cys**	UGU	112	0.33	**0**	69	0.21	**0**	40	0.12	**0**
**Cys**	**UGC**	231	0.67	**2**	260	0.79	**2**	296	0.88	**1**
										
**Gln**	**CAG**	508	0.71	2	604	0.86	4	653	0.95	3
**Gln**	CAA	209	0.29	1	102	0.14	2	35	0.05	1
										
**Glu**	**GAG**	893	0.67	2	1084	0.8	4	1183	0.93	2
**Glu**	GAA	441	0.33	1	266	0.2	1	93	0.07	1
										
**Gly**	GGG	176	0.11	1	177	0.12	2	111	0.08	1
**Gly**	GGA	246	0.15	1	163	0.11	2	43	0.03	1
**Gly**	GGU	750	0.46	**0**	362	0.24	**0**	304	0.21	**0**
**Gly**	**GGC**	469	0.29	**3**	829	0.54	**4**	997	0.69	**4**
										
**His**	CAU	111	0.28	**0**	73	0.18	**0**	52	0.13	**0**
**His**	**CAC**	292	0.72	**1**	344	0.82	**4**	349	0.87	**2**
										
**Ile**	AUA	82	0.08	1	37	0.04	3	20	0.02	1
**Ile**	AUU	430	0.42	**2**	350	0.38	**4**	165	0.18	**3**
**Ile**	**AUC**	516	0.5	**0**	533	0.58	**0**	753	0.8	**0**
										
**Leu**	UUG	243	0.14	0	154	0.09	2	72	0.05	1
**Leu**	UUA	61	0.04	1	16	0.01	2	6	0	1
**Leu**	**CUG**	526	0.31	1	764	0.46	2	970	0.64	2
**Leu**	CUA	107	0.06	1	39	0.02	2	29	0.02	1
**Leu**	CUU	391	0.23	**2**	343	0.21	**4**	138	0.09	**3**
**Leu**	CUC	375	0.22	**0**	336	0.2	**0**	289	0.19	**0**
										
**Lys**	**AAG**	1248	0.78	3	1458	0.9	4	1457	0.96	3
**Lys**	AAA	350	0.22	1	158	0.1	2	66	0.04	1
										
**Met**	AUG	571	1	3	558	1	6	596	1	4
										
**Phe**	UUU	261	0.35	**0**	327	0.43	**0**	111	0.16	**0**
**Phe**	**UUC**	479	0.65	**2**	426	0.57	**4**	579	0.84	**2**
										
**Pro**	**CCG**	191	0.22	1	290	0.33	2	469	0.59	2
**Pro**	CCA	190	0.22	1	138	0.16	2	63	0.08	1
**Pro**	CCU	185	0.22	**1**	152	0.18	**2**	75	0.09	**2**
**Pro**	CCC	288	0.34	**0**	287	0.33	**0**	187	0.24	**0**
										
**Ser**	AGU	137	0.11	**0**	77	0.06	**0**	39	0.03	**0**
**Ser**	AGC	234	0.19	**2**	299	0.25	**2**	330	0.28	**2**
**Ser**	**UCG**	210	0.17	1	302	0.25	2	384	0.32	1
**Ser**	UCA	180	0.14	1	85	0.07	2	35	0.03	1
**Ser**	UCU	237	0.19	**1**	158	0.13	**2**	132	0.11	**1**
**Ser**	UCC	262	0.21	**0**	274	0.23	**0**	276	0.23	**0**
										
**Thr**	**ACG**	368	0.29	1	553	0.47	2	622	0.58	2
**Thr**	ACA	328	0.26	1	182	0.16	2	82	0.08	1
**Thr**	ACU	280	0.22	**1**	141	0.12	**2**	74	0.07	**3**
**Thr**	ACC	276	0.22	**0**	292	0.25	**0**	294	0.27	**0**
										
**Trp**	UGG	194	1	1	204	1	2	167	1	1
										
**Tyr**	UAU	177	0.31	**0**	83	0.15	**0**	39	0.07	**0**
**Tyr**	**UAC**	391	0.69	**1**	466	0.85	**2**	510	0.93	**3**
										
**Val**	**GUG**	805	0.47	1	1022	0.6	4	1115	0.69	2
**Val**	GUA	200	0.12	1	60	0.04	2	46	0.03	1
**Val**	GUU	449	0.26	**2**	318	0.19	**2**	141	0.09	**2**
**Val**	GUC	250	0.15	**0**	290	0.17	**0**	322	0.2	**0**

Although all two-fold degenerate codons show preference for GC3, this feature is not universal throughout the high-expression gene sets. *T. brucei *shows little bias for Ala, Pro, Ser or Thr codons and GGG^Gly^, AGG^Arg ^and CGG^Arg ^codons are more than two-fold under-represented in all three trypanosomatids (Fig. 1 and Table 1).

### 'Favoured' codons correspond to over-represented cognate tRNAs

Biased codons likely favour translation if cognate tRNAs are more abundant [7,23]. Previous analysis indicated that *T. brucei *tRNA genes are organised into clusters spread over several chromosomes and that relative tRNA abundance correlates with codon usage but not with gene copy number [24]. It was noted in that study, however, that tRNA nucleotide modification may lead to under-estimation of tRNA abundance in some cases. The tRNA gene complement was analysed in all three trypanosomatids in order to explore the relationship between codon usage and tRNA gene copy number.

The GeneDB database revealed a total of 261 annotated tRNAs among the three trypanosomatids. The universal genetic code comprises 61 codons for 20 amino acids but some tRNAs decode multiple codons, a phenomenon known as wobble [25]. The trypanosomatid tRNA complement (see table 2 in the additional data file) represents 45 anticodons, a tRNA gene distribution that suggests that sixteen CU3 codons are decoded by wobble tRNAs (see Table 1). Eight U3 codons are likely decoded by anticodons with guanosine while another eight C3 codons are likely decoded by anticodons with inosine (deaminated adenosine) in the wobble position respectively [25,26]. All but one of these 'wobble pairs' were predicted previously in *T. brucei *[24]. Codon bias is seen among wobble pairs in the high-expression gene set; Asn, Asp, Cys, His, Phe and Tyr for example (Table 1), possibly reflecting translational selection based on differential stability of codon-anticodon interaction. In addition, some wobble codon pairs and putative cognate tRNAs are biased and over-represented respectively; CGU/C^Arg^-ACG^tRNA ^and GGU/C^Gly^-GCC^tRNA ^for example (Table 1).

**Figure 2:**
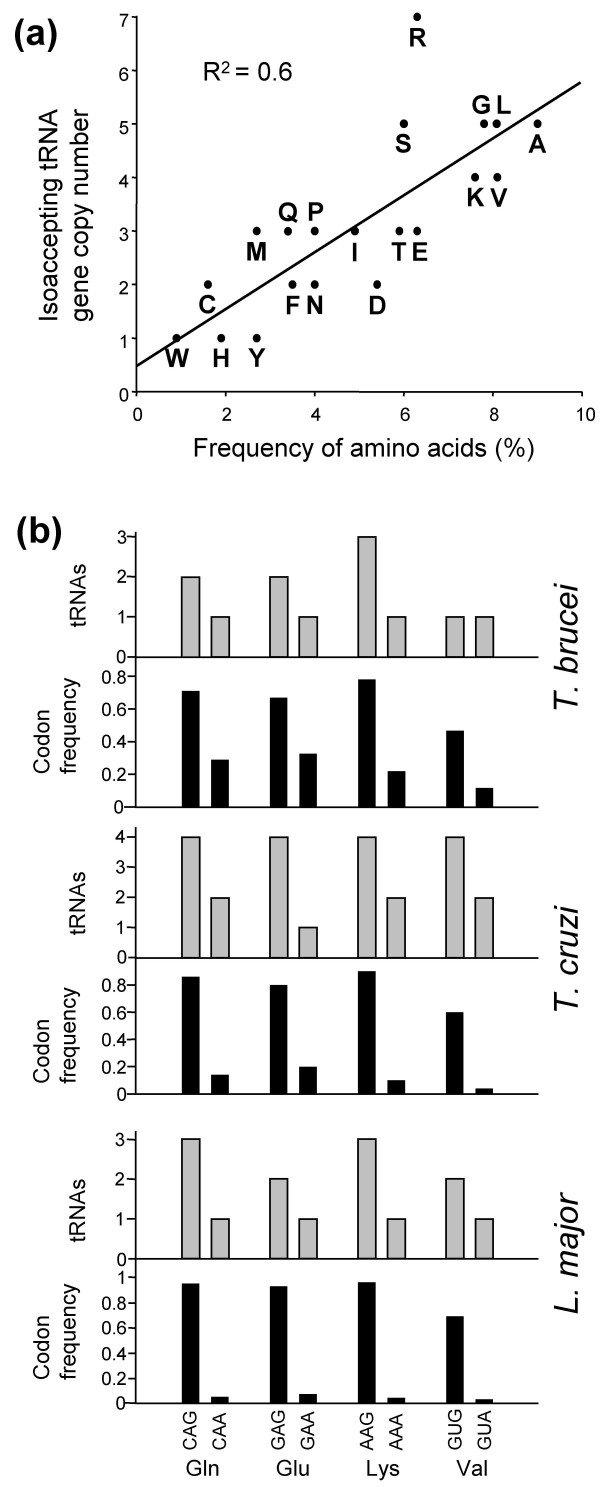
Correspondence between codon-usage in highly expressed genes and tRNA gene copy number. **(a) **Correspondence between amino acid frequency and cognate tRNA gene copy number in *T. brucei*; patterns were broadly similar in *T. cruzi *and *L. major *(data not shown). **(b) **Correspondence between synonymous codon usage (lower charts with black bars) and cognate tRNA gene copy number (upper charts with grey bars). The GA3 codon-pairs with >30% bias in all three trypanosomatids are shown.

To test the idea that tRNA abundance is related to gene copy number in trypanosmatids, amino acid frequency in the tandem, high-expression protein set was plotted against tRNA gene copy number (Fig. 2A). The positive correlation strongly supports the idea that tRNA gene copy number determines relative tRNA abundance. Having established this relationship, the four GA3 synonymous codon-pairs with >30% bias in all three trypanosomatids were analysed (Fig. 2B). The results show a positive correlation between tRNA gene copy number and codon usage bias providing further evidence for translational selection; only one *T. brucei *tRNA gene fails to display a matching bias in copy number. The correlation is more striking in *L. major*, the trypanosomatid that displays more pronounced codon bias. In this case, eight of nine GA3 pairs that display substantial codon bias also display a corresponding tRNA bias (Table 1; those shown in Fig. 2b plus GCG/A^Ala^, CUG/A^Leu^, CCG/A^Pro ^and ACG/A^Thr^). Indeed, there are several examples in *Leishmania *where tRNA genes that recognise favoured codons appear to have been specifically amplified since divergence from the trypanosomes (CAG^Gln^, CUG^Leu^, CCG^Pro^, ACG^Thr ^and favoured 'wobble' codons; CGC^Arg^, GGC^Gly^, AUC^Ile^, CUC^Leu^, CCC^Pro ^and ACC^Thr^; see Table 1), perhaps to counter the high background GC-content. Thus, there is a correspondence between numbers of cognate tRNAs, a likely measure of tRNA abundance, and preferred codons in the high expression gene sets in all three trypanosomatid genomes.

### Codon usage correlates with expression level

**Figure 3:**
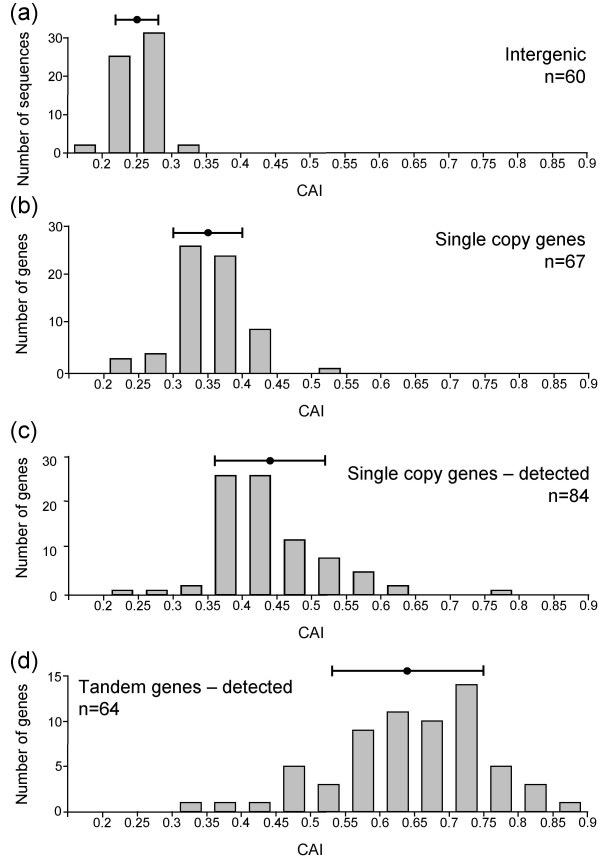
Codon usage correlates with expression level. *T. cruzi *sequences were divided into four categories and CAI scores were calculated. The distribution of scores is indicated for each category. **(a) **intergenic regions (translated in all six reading frames). **(b) **'Single-copy' genes (see table 4 in the additional data file). **(c) **'Single-copy' genes detected (≥ 5 mass spectra) through whole-cell proteome analysis (see table 3 in the additional data file). **(d) **Tandem-arrayed genes detected (≥ 10 mass spectra) through whole-cell proteome analysis (see table 1 in the additional data file). The average score +/- standard deviation is indicated for each category.

The codon adaptation index (CAI) is used to measure synonymous codon usage bias and can predict gene expression level if translational selection operates [27]. Whole-cell proteome data are available for *T. cruzi *[18] and the number of mass-spectra matched to individual genes provides an indication of relative expression level. Thus, proteome data provide an opportunity to test for a correlation between codon usage and expression. For this analysis, *T. cruzi *sequences were divided into four categories expected to have progressively higher CAI scores if translational selection operates. The categories were as follows: (a) intergenic regions (translated in all six reading-frames); (b) 'Single-copy' genes; (c) 'Single-copy' genes detected through whole-cell proteome analysis (≥ 5 mass spectra) and (d) Tandem-arrayed genes detected through whole-cell proteome analysis (≥ 10 mass spectra). The analysis shown in Figure 3 indicates progressively increasing CAI scores from (a) through (d). Protein coding sequences appear to have evolved to optimise translation while intergenic sequences may have evolved to counter translation.

### Codon usage can predict expression level for individual proteins

**Figure 4:**
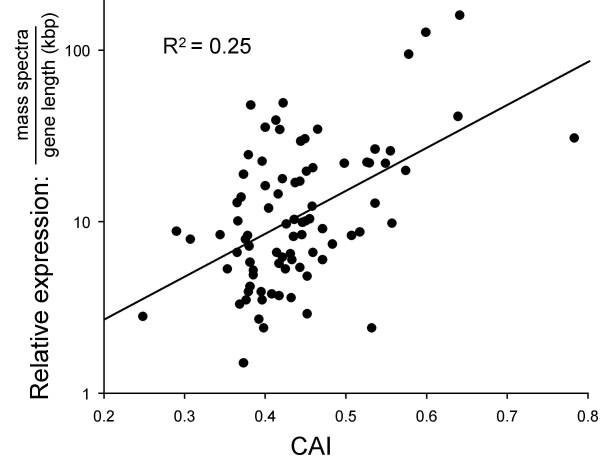
Codon usage is predictive of expression level for individual genes. CAI scores were calculated for single-copy genes (see Fig. 3c and table 3 in the additional data file). The number of mass spectra, corrected for gene length, provides a measure of relative abundance. Only genes represented by ≥ 5 mass spectra from *T. cruzi *proteome analysis [21] were analysed to minimise sampling errors.

A more rigorous test of the contribution of translational selection to gene expression is whether expression level can be predicted for individual genes exclusively based on codon usage. Single-copy genes represented in the *T. cruzi *proteome data [21] were used for this analysis (see table 3 in the additional data file); tandem array genes were not suitable because gene copy number, thought to contribute to expression, is highly variable and unknown in most cases. CAI scores were calculated for each gene and plotted against the gene length-adjusted number of cognate mass-spectra, a measure of relative expression. A positive correlation emerges and the relationship between protein abundance and CAI is log-linear (Fig. 4). The results indicate that *T. cruzi *protein-coding sequences can predict relative steady-state protein expression level. The trend is remarkable because of the range of other parameters, both alternative modes of expression control and experimental sampling that could impact on the outcome. The findings are consistent with the idea that codon bias has a major impact on steady state protein levels in trypanosomatids.

### Relative codon bias is conserved among trypanosomatids

**Figure 5:**
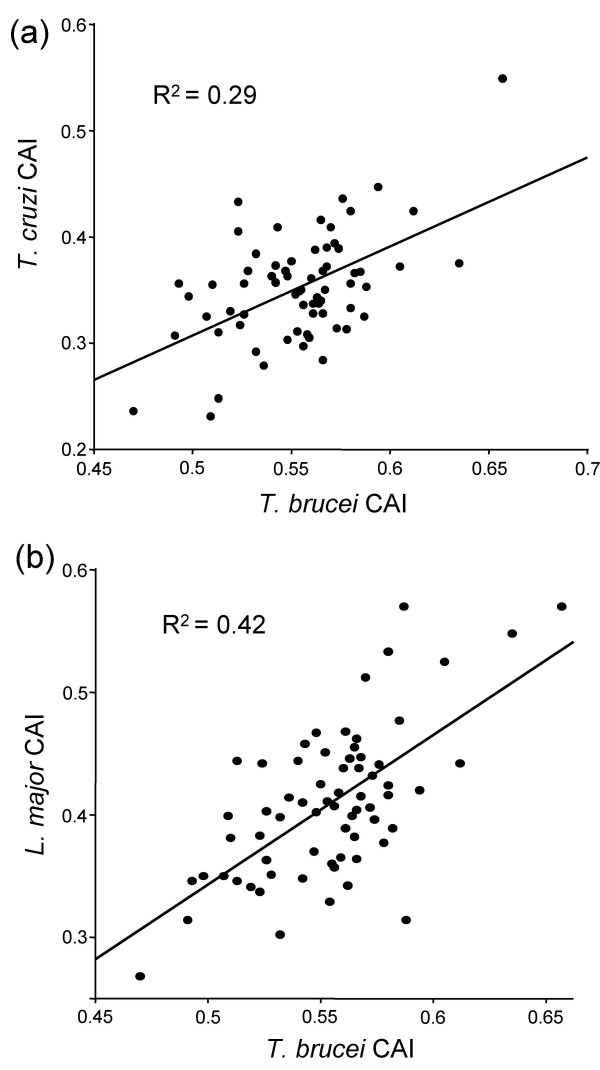
Relative codon bias is conserved among trypanosomatids. CAI scores were calculated for single-copy, protein-coding sequences. **(a) **Relative scores for *T. brucei *and *T. cruzi*. **(b) **Relative scores for *T. brucei *and *L. major*. The syntenic, polycistronic clusters analysed were from, *T. brucei *chromosomes 1, 6 and 10 and from the orthologous genes in *T. cruzi *and on *L. major *chromosomes 12, 30 and 3 (see table 4 in the additional data file). The *T. cruzi *set is the same as that presented in Figure 3b; the chromosome numbers have not been determined in *T. cruzi *due to problems with sequence assembly.

Current, high throughput technologies fail to detect and/or quantify the expression levels of less abundant proteins [28]. An interesting question is whether CAI can predict expression level across the genome. Although certain proteins will be required in substantially different quantities in the different trypanosomatids, the majority are expected to be expressed at similar relative levels. Thus, relative CAI scores are expected to be broadly conserved if translational selection impacts upon global gene expression. CAI scores were calculated for 'single-copy' genes from syntenic, polycistronic gene clusters on three different chromosomes (see table 4 in the additional data file). The different gene clusters from each trypanosomatid showed similar CAI distribution so the data were pooled. CAI scores were first compared in the trypanosomes, *T. brucei *and *T. cruzi *and the analysis indicates that relative scores are indeed conserved (Fig. 5A). A more rigorous test was then carried out, between *T. brucei *and *L. major*, thought to have diverged around 250 Mya. Despite the substantially higher GC content in *L. major*, relative scores remain broadly conserved (Fig. 5B). The results are consistent with the idea that codon bias predicts translation efficiency for any mRNA in trypanosomatids.

## Discussion

In trypanosomatids, bias in codon usage correlates with tRNA gene copy number and with expression level. This provides strong evidence for a major impact of translational selection on gene expression. Thus, translational selection facilitates the generation of differential protein abundance from genes embedded within polycistrons. Since translation rates are likely retarded by codons with low-abundance cognate tRNAs, natural selection of tRNA gene numbers and codon bias allows optimization of translation rate and efficiency across the genome. Many of the most highly expressed genes use a dual strategy to enhance expression; increased gene dosage combined with a high proportion of codons with more abundant cognate tRNAs. This dual strategy allows for an increase in overall transcription and translation.

In *S. cerevisiae*, although the value of codon bias as a predictor of protein levels is disputed, proteins encoded by genes with low bias are not detected on two-dimensional gels and protein abundance does correlate when only genes with high bias are considered [29,30]. Thus, translational selection may be a pervasive mechanism in the control of gene expression but its impact may be obscured in many cell-types due to the impact of other regulatory mechanisms. I propose that translational selection makes a more substantial contribution to gene expression control in trypanosomatids due to the paucity of regulated transcription. Initial ribosome assembly on mRNA may also be largely unregulated since trans-splicing leads to the attachment of an identical spliced-leader sequence to every mRNA [4]. Thus, differential translation efficiency may be the dominant level of gene expression control in trypanosomatids. Translational selection may have emerged in primitive cells that lacked mechanisms for differential mRNA expression and the emergence of differential transcription in other cell types may have obscured or partially replaced this mode of control.

Many trypanosomatid proteins are differentially expressed during the cell-cycle and the life-cycle and additional controls must clearly determine such differential expression. A number of mRNA un-translated regions, particularly at the 3' end, may modulate mRNA maturation, transport, turnover and translation for example and protein turnover may also vary [4]. When these additional controls operate, codon bias should fail to predict expression level. Prominent examples of differential regulation include the variant surface glycoprotein gene, abundantly expressed in bloodstream form *T. brucei*, and procyclins, expressed in insect stage *T. brucei*. Expression of these proteins is regulated using an unusual mechanism involving differential transcription by RNA polymerase I which is restricted to *RRNA *genes in other eukaryotes [31]. mRNA turnover [32] and protein turnover [33] also contribute to controlling variant surface glycoprotein expression and, as expected, codon bias fails to predict relative expression when these controls operate (CAI for variant surface glycoprotein genes = 0.54 +/-0.01. n = 4). Thus, codon analysis in combination with high-throughput proteome analysis may allow identification of proteins subject to the alternative expression control strategies described above. In addition, orthologous genes that show poor correspondence in relative codon bias among trypanosomatids may be those that display species-specific expression differences. If this is the case, genome-wide codon-usage analysis will facilitate the identification of these genes.

Protein coding sequences are relatively easy to predict in trypanosomatids due to high density, intron poverty and organisation into directional clusters. New annotation tools are under development, however [34], and gene annotation could be refined. The findings reported here indicate that algorithms incorporating codon sampling could facilitate the annotation of current and future trypanosomatid genome sequence data.

## Conclusion

Constitutive RNA polymerase II transcription is widespread in trypanosomatids so differential gene expression must be controlled post-transcription. Research in this area has focussed on un-translated mRNA regulatory sequences typically found within 3' un-translated regions. As reported here, analysis of synonymous codon bias indicated pronounced bias in highly expressed genes and this bias correlates with tRNA gene copy number and with gene expression level. In addition, relative codon bias is conserved among orthologous genes from divergent trypanosomatids, even in genes thought to be expressed at low level. Taken together, the results suggest that control at the level of translation, translational selection, is the dominant mechanism underlying differential protein expression in these organisms.

## Methods

### Analysis of sequence and expression data

Annotated trypanosomatid genome sequence data were browsed and analysed using the GeneDB interface [35] hosted by the Wellcome Trust Sanger Institute [36]. *T. cruzi *proteome expression data [21] were obtained from Supporting Online Material available through the *Science *website [37]. Codon usage was determined using the Codon Usage feature [38] within The Sequence Manipulation Suite [39]. CodonW [40] was used to generate codon usage tables for each trypanosomatid and to calculate CAI scores. Codon usage tables were assembled using fifty genes from each tandem, high expression gene set (Table 1 in the additional data file). Data were manipulated and analysed using Excel (Microsoft).

## List of abbreviations

CAI = Codon Adaptation Index.

## References for additional file

1. **GeneDB **.

Table S1 and Table S3

2. Atwood JA, 3rd, Weatherly DB, Minning TA, Bundy B, Cavola C, Opperdoes FR, Orlando R, Tarleton RL: **The *Trypanosoma cruzi *proteome**. *Science *2005, **309**(5733):473–476.

Table S1

3. Mottram JC, Murphy WJ, Agabian N: **A transcriptional analysis of the *Trypanosoma brucei hsp83 *gene cluster**. *Mol Biochem Parasitol *1989, **37**(1):115–127.

4. Marchand M, Poliszczak A, Gibson WC, Wierenga RK, Opperdoes FR, Michels PA: **Characterization of the genes for fructose-bisphosphate aldolase in *Trypanosoma brucei***. *Mol Biochem Parasitol *1988, **29**(1):65–75.

## Supplementary Material

Additional file 1 All genes listed are linked to the GeneDB database [36]. The colour coding in additional tables S1, S3 and S4 is based on the GeneDB annotation. *T. cruzi *mass spectra values in additional tables S1 and S3 are from Atwood et al., [21]. Table S1 – Trypanosomatid tandem/high-expression set. Only the *T. brucei *GeneDB links are listed but further links to orthologous trypanosomatid genes can be found on each GeneDB page. Grey shading represents genes thought to be present in tandem arrays but not annotated (An) as such; HSP83 [41] and aldolase [42] in *T. brucei *for example, but mostly due to incomplete assembly of *T. cruzi *genome sequence. Table S2 – Trypanosomatid tRNA genes. tRNA genes and cognate codons are indicated. Table S3 – *T. cruzi *'single-copy' gene analysis. All these genes have orthologues annotated in the other trypanosomatids and are represented by ≥ 5 cognate mass spectra in the non-redundant proteome set. Table S4 – Realtive CAI analysis for three polycistronic gene clusters. The single-copy genes analysed are from: i. The 75 kbp polycistronic gene cluster on *T. brucei *chromosome 1 (GeneDB coordinates 815 – 985 kbp), syntenic with the 375 kbp cluster on *L. major *chromosome 12 (GeneDB coordinates 295 – 670 kbp). ii. The 120 kbp polycistronic gene cluster on *T. brucei *chromosome 6 (GeneDB coordinates 1,285 – 1,405 kbp), syntenic with the 170 kbp cluster on *L. major *chromosome 30 (GeneDB coordinates 1,225 – 1,395 kbp). iii. The 85 kbp polycistronic gene cluster on *T. brucei *chromosome 10 (GeneDB coordinates 1,140 – 1,225 kbp), syntenic with the 130 kbp cluster on *L. major *chromosome 36 (GeneDB coordinates 20 – 150 kbp). The syntenic chromosome numbers have not been determined in *T. cruzi *due to problems with sequence assembly. Click here for file
